# The Hospitals of New South Wales

**Published:** 1905-11-11

**Authors:** 


### THE HOSPITALS OF NEW SOUTH WALES.

The needs of the hospitals have recently been claiming the attention of the Legislative Assembly of New South Wales. So far back as April 1904 a report was submitted by Mr. George McRae, of the Government Architect's Office, regarding the necessity for the addition of new nurses' quarters and an isolation ward to the Lower Clarence Hospital at Maclean. The combined cost of these buildings was estimated at £1,375. In September of this year the matter again came up for discussion by the Legislative Assembly, and the Government was asked to "consider as early as possible the necessity of granting a sum of money on condition that the Hospital Committee provided half the cost of the necessary improvements." Several of the country hospitals, among them the Gundagai District Hospital, are suffering from financial difficulties, owing to the non-payment of subsidies, and are being worked under large overdrafts on the banks. The matter was promised attention as soon as funds were available.

